# Case report: One case of umbilical vein thrombosis in the second trimester with associated portal vein thrombosis after childbirth

**DOI:** 10.3389/fmed.2023.1281896

**Published:** 2023-12-06

**Authors:** Wei-Wei Dai, Qi-Shu Hou, Lian-Hua Yang, Shang-Qin Chen, Ji-Feng Ye

**Affiliations:** ^1^Department of Pharmacy, the Second Affiliated Hospital and Yuying Children’s Hospital of Wenzhou Medical University, Wenzhou, China; ^2^Department of Neonatology, the Second Affiliated Hospital and Yuying Children’s Hospital of Wenzhou Medical University, Wenzhou, China

**Keywords:** neonate, umbilical vein thrombosis, portal vein thrombosis, prenatal ultrasound, fetal outcomes

## Abstract

**Background:**

Umbilical vein thrombosis is a rare pregnancy complication, that is difficult to detect prenatally but can lead to poor fetal outcomes.

**Case presentation:**

We described a 33-year-old primiparae who was identified as having umbilical vein thrombosis by ultrasound at 21 weeks gestation, and the neonate was found to have a portal vein thrombus after delivery. Following enoxaparin anticoagulant therapy, the thrombus disappeared within 4 weeks. No thrombus formation occured during the 10-month follow-up, and the baby was in excellent clinical condition.

**Conclusion:**

Owing to the poor fetal outcomes related to umbilical thrombosis, pay attention to abnormal clinical signs during prenatal ultrasound, fetal heart monitoring and counting fetal movements can help in the early identification of umbilical cord thrombosis.The findings highlight the importance of regular prenatal ultrasound evaluation, enabling early detection and monitoring of any anomalies or vascular abnormalities related to the fetal umbilical vein. Further research is warranted to explore the clinical implications and long-term outcomes associated with these findings.

## Background

Thrombosis of the umbilical cord vessels is a rare event, occurring in 1 in 1500 deliveries, which can lead to poor fetal outcomes (1). Thrombi involving the umbilical cord are more frequently seen in the vein, even if adverse pregnancy outcomes are more associated with arterial thrombosis (2). Umbilical vein thrombosis (UVT) is difficult to detect during routine antenatal examinations, so its diagnosis and treatment remain a challenge. In addition, there was only one case report to describe portal vein thrombosis in the context of umbilical vein thrombosis, which was published in 2008 (3). Here, we report a rare case of umbilical vein thrombosis (UVT) in a neonate that was detected prenatally via ultrasound examination, and portal vein thrombosis was identified after birth.

## Case presentation

**Figure 1:**
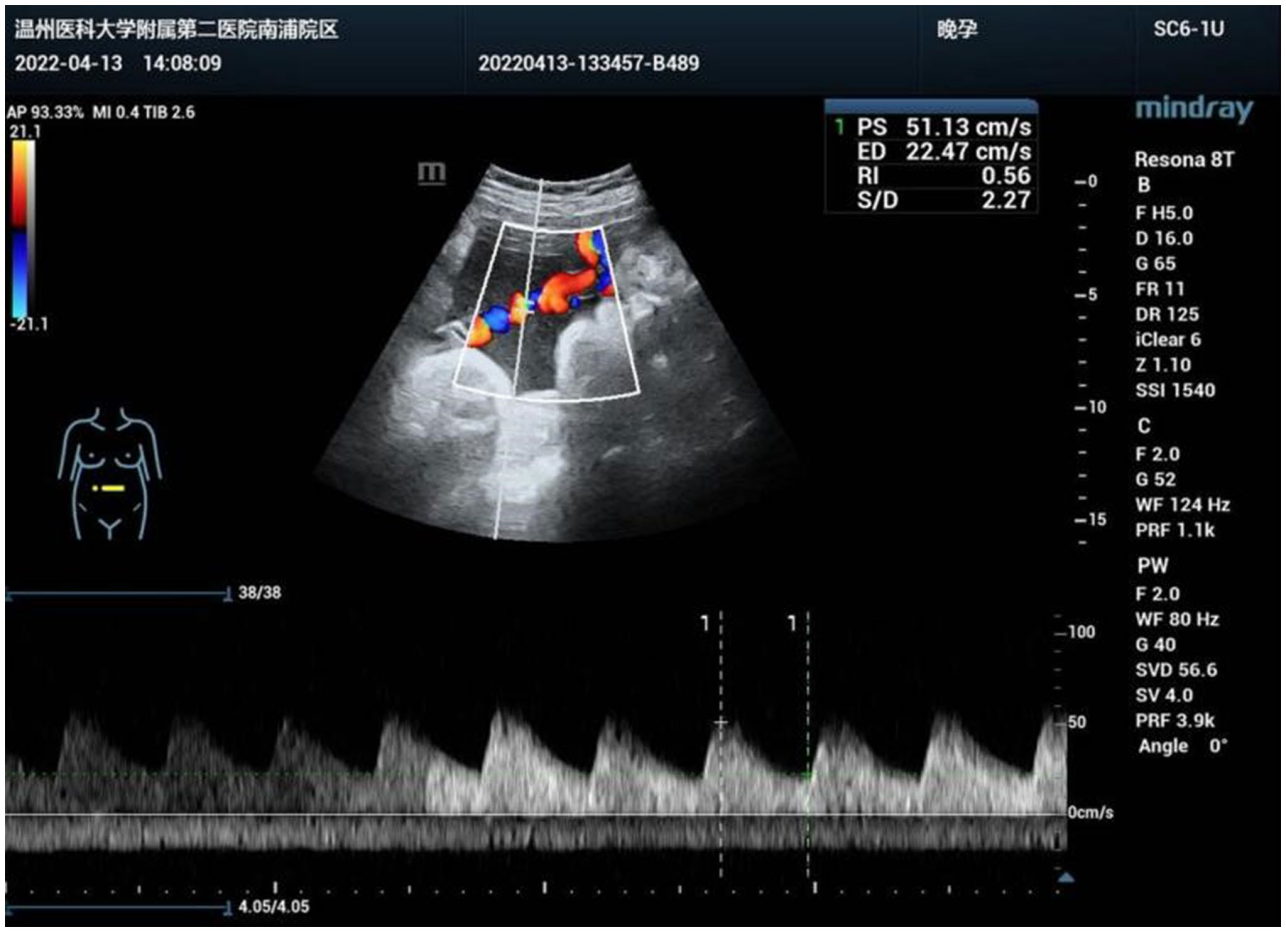
Abnormal bean-like dilation of the intra-abdominal umbilical vein suggestive of umbilical vein thrombosis at 21 weeks of gestation.

**Figure 2:**
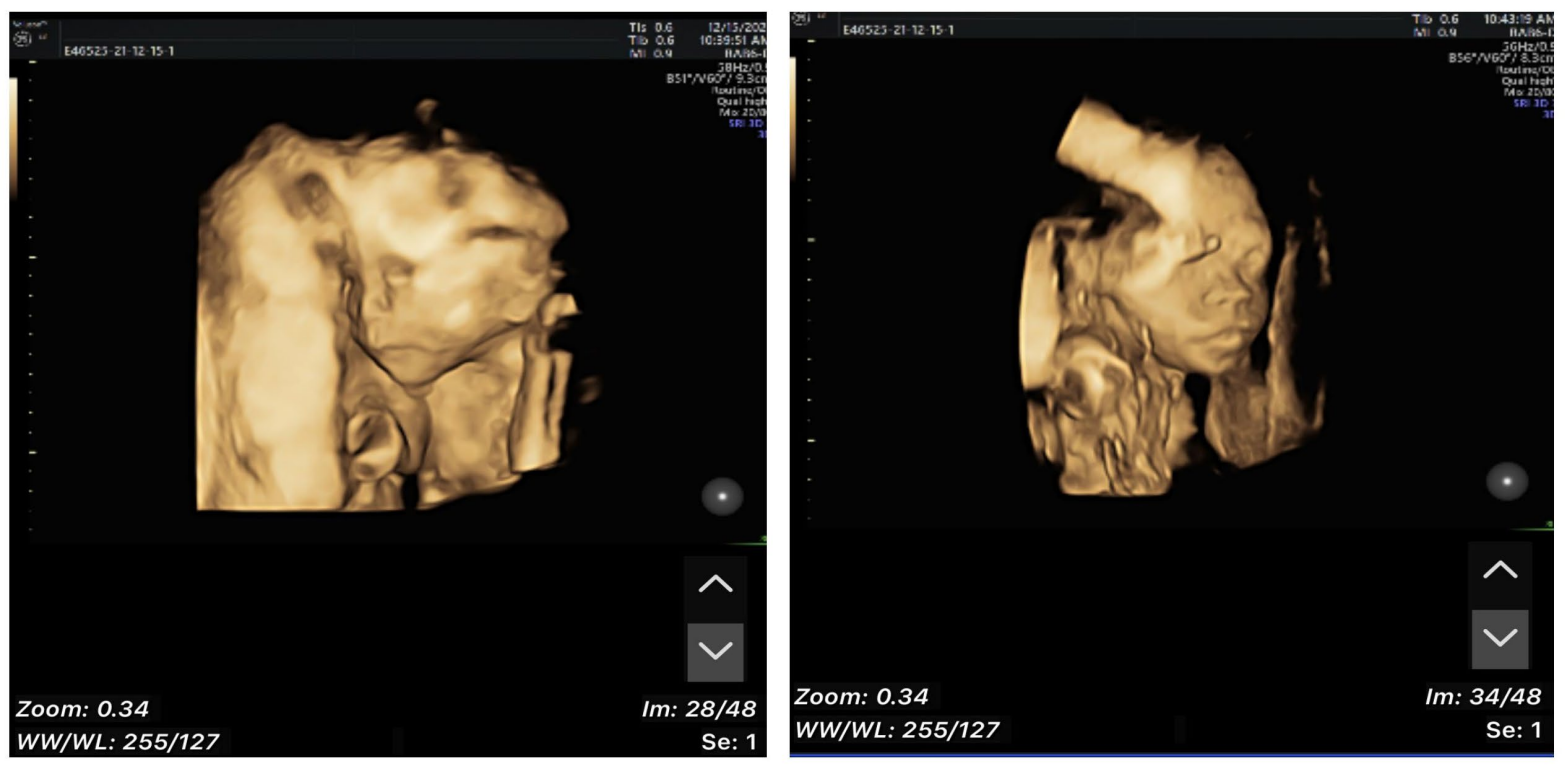
Fetal color ultrasound image at 21 weeks.

A 33-year-old primiparae was admitted in spontaneous labor to the labor and delivery service of the Second Affiliated Hospital of Wenzhou Medical University (Wenzhou, China) at 40 weeks of gestation. The patient was in good health during pregnancy, except for gestational diabetes mellitus. She had a routine obstetrical ultrasound at 21 weeks gestation, which revealed an abnormal bean-like dilation of the intra-abdominal umbilical vein, and umbilical vein thrombosis was suspected (Figure 1). Table 1 shows the umbilical vein flow pattern from 21 weeks to 40 weeks. Figure 2 shows the fetal color ultrasound image at 21 weeks. The fetal system ultrasound examination was performed at 21 weeks gestation and showed no abnormalities in the fetal biometric measurements, head, facial features, spine, chest, heart, diaphragm, stomach, abdominal wall, kidneys, bladder, forearm bones, lower leg bones, hands, feet, and bilateral adnexal regions of the pregnant woman. The placenta was located on the posterior wall of the uterus with normal thickness and grade, and the maximum depth of amniotic fluid pool was within normal range. The umbilical cord had a normal morphology with two umbilical arteries and one umbilical vein, and normal blood flow waveform in the umbilical artery. There was a local dilation of the intra-abdominal segment of the umbilical vein, but with normal blood flow parameters. Subsequently, she was scheduled for close follow-up with prenatal ultrasound every 2 weeks. Repeat ultrasound examination showed that the hyperechoic oval structure enlarged gradually, but there were no obvious abnormalities in umbilical cord blood flow, fetal growth or fetal heart monitoring. The delivery was uneventful, and an apparently healthy female infant weighing 2,830 grams was delivered. Apgar scores were 10 at 1 min and 10 at 5 min. The amniotic fluid was clear, and the placenta was delivered intact. No gross placental lesions or umbilical cord anomalies were identified. An abdominal ultrasound was performed soon after birth, which suggested thrombosis in the umbilical vein and portal vein. The baby was subsequently transferred to the neonatal intensive care unit (NICU) for clinical monitoring and observation.

**Table 1:** The umbilical vein flow pattern from 21 weeks to 40 weeks.

Gestational Age	Date	Umbilical vein blood flow
21 weeks	2021.12.15	Intra-abdominal segment: locally dilated, spindle-shaped
		Blood flow signal: swirling
		Widest diameter: approximately 11 mm
		Liver segment internal diameter: approximately 4 mm
		Abdominal wall segment diameter: approximately 2 mm
		Fetal umbilical vein: localized tumor-like dilation
24 weeks	2022.01.05	Intra-abdominal segment: locally dilated, spindle-shaped
		Blood flow signal: swirling
		Widest diameter: approximately 14.6 mm
		Left branch of portal vein internal diameter: approximately 5.3 mm
		Abdominal wall diameter: approximately 2.9 mm
		Fetal umbilical vein: localized tumor-like dilation
27 weeks	2022.01.26	Intra-abdominal segment: locally dilated, spindle-shaped
		Blood flow signal: swirling
		Widest diameter: approximately 16 mm
		Left branch of portal vein internal diameter: approximately 5.8 mm
		Abdominal wall diameter: approximately 2.9 mm
		Fetal umbilical vein: localized tumor-like dilation
30 weeks	2022.02.16	Intra-abdominal segment: locally dilated, cystic
		Size: approximately 24 × 29mm
		Blood flow signal: swirling
		Fetal umbilical vein in the liver: tumor-like dilation
33 weeks	2022.03.08	Intra-abdominal segment: locally dilated, cystic
		Size: approximately 24 × 31 × 26mm
		Blood flow signal: swirling
		Flow velocity: 1.35 m/s
		Fetal umbilical vein in the liver: tumor-like dilation
36 weeks	2022.03.26	Intra-abdominal segment: locally dilated, spindle-shaped
		Blood flow signal: swirling
		Widest diameter: approximately 25 mm
		Left branch of portal vein internal diameter: approximately 7.9 mm
		Abdominal wall diameter: approximately 5.1 mm
		Fetal umbilical vein: localized tumor-like dilation
38 weeks	2022.04.13	Intra-abdominal segment: locally dilated, cystic
		Size: approximately 48 × 27mm
		Blood flow signal: swirling
		Fetal umbilical vein: localized tumor-like dilation

**Figure 3:**
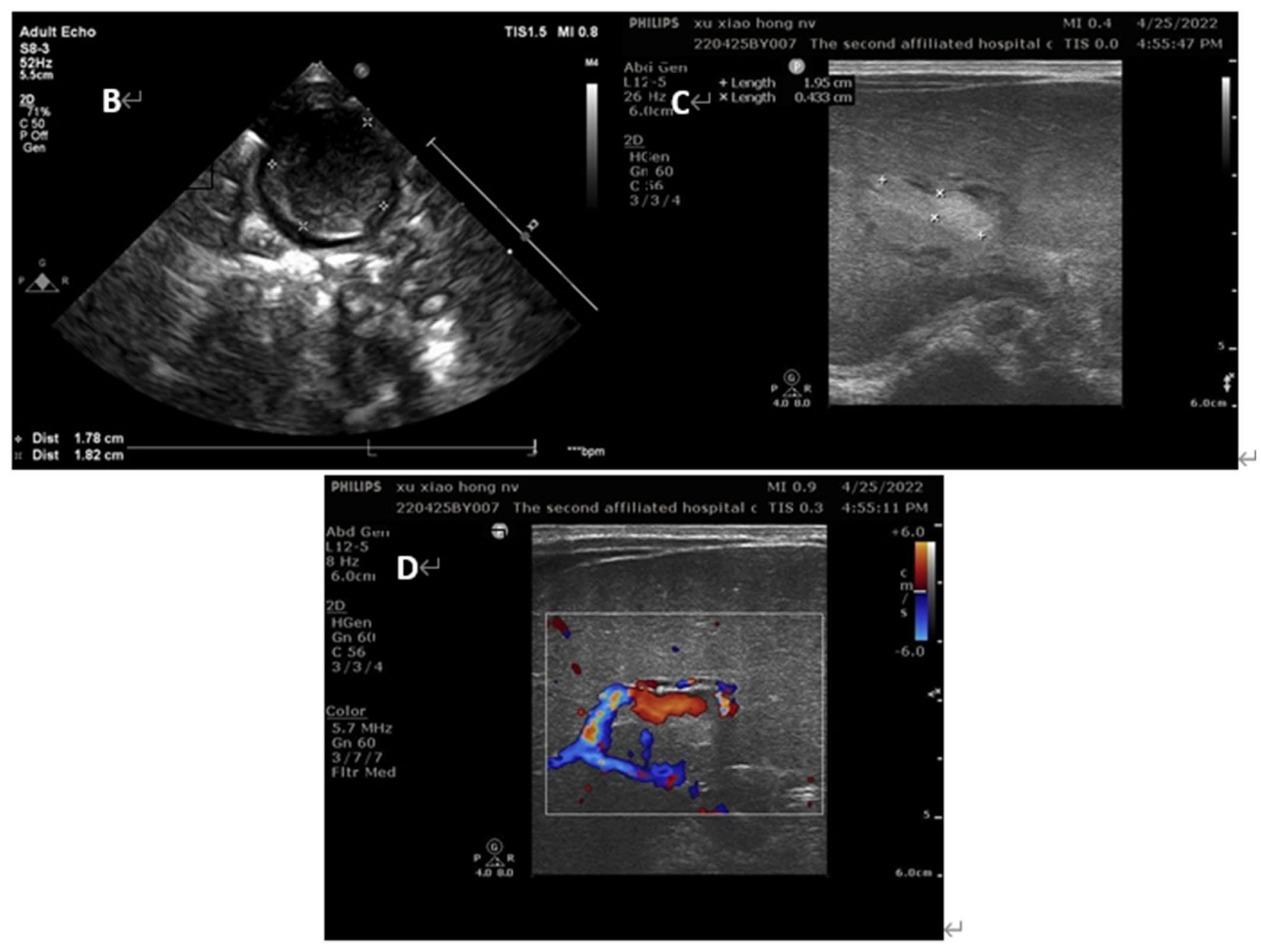
Ultrasound images of the patients on day 2 of life **(B)** umbilical vein thrombus at day 2; **(C)** portal vein thrombus at day 2; **(D)** the blood flow signal decreased in the lumen of portal vein thrombosis at day 2.

Her vital signs were normal, and her feeding was uneventful. On the second day of life, a second abdominal ultrasound confirmed the presence of a thrombus of 26 × 16 mm in umbilical vein thrombosis and a thrombus of 21 × 5 mm in portal vein (Figure 3). Fortunately, there was no thrombosis in the renal vein, splenic vein, superior mesenteric vein or inferior vena cava. Subsequent cerebral ultrasound excluded intracranial thrombosis. The laboratory examination made on admission found, white blood cells at 14.21× 10^9^/L, hemoglobin at 175 g/L, platelets at 183× 10^9^/L, and C-reactive protein (CRP) at 13.65 mg/L. Prothrombotic states were also evaluated: Protein C 26%, antithrombin III 48%, lupus ratio 0.58, d-dimer 2.38 μg/mL, and other indicators were within normal ranges.

**Figure 4:**
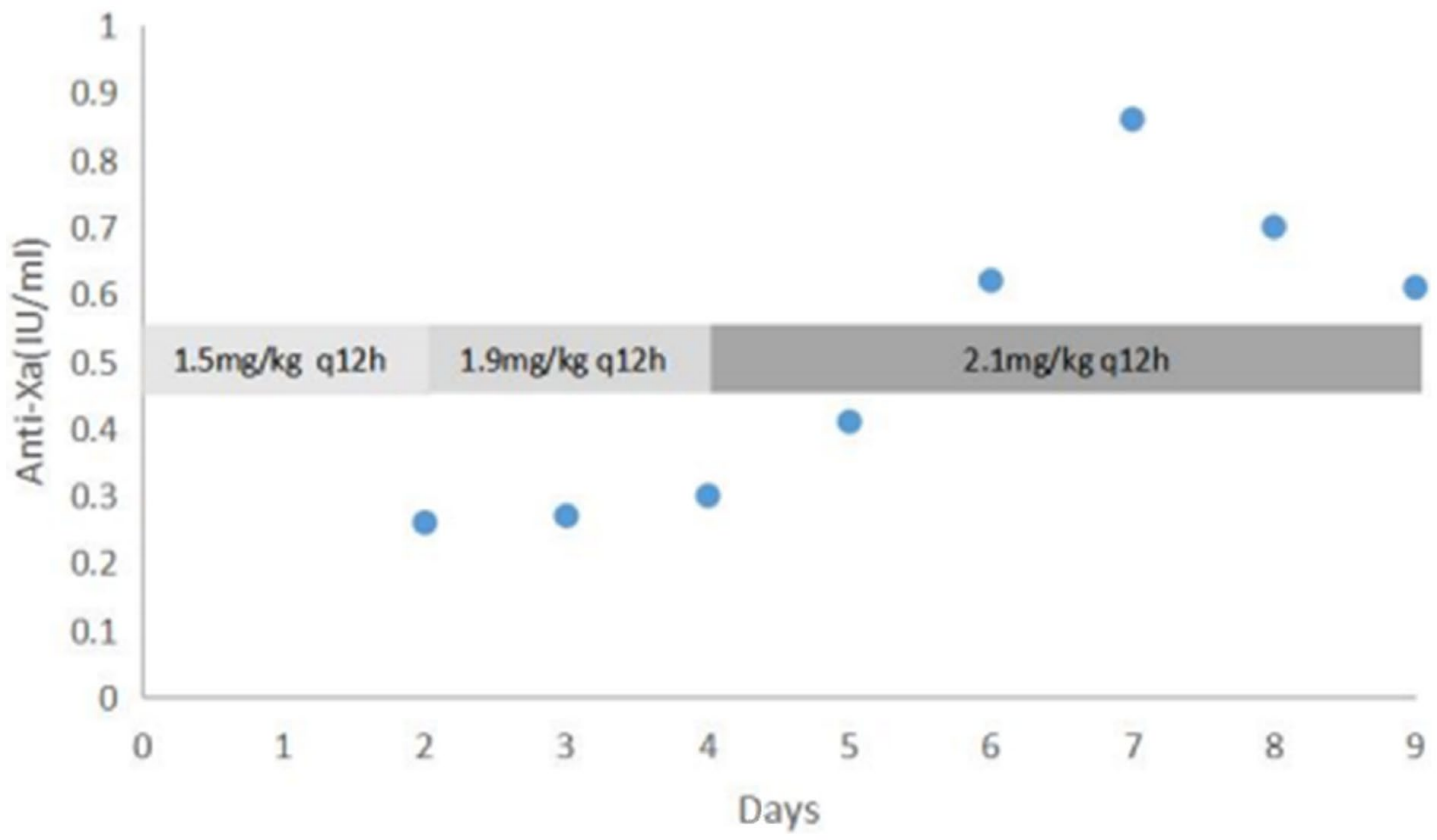
Dose adjustment of enoxaparin according to anti-Xa level (IU/mL) Days refers to the duration of enoxaparin treatment.

According to American College of Chest Physicians guidelines, anticoagulation with low-molecular weight heparin (enoxaparin) at a dose of 1.5 mg/kg twice a day was started at day of life 3, targeting an anti-Xa between 0.5 and 1.0 IU/mL (4). A third ultrasound performed at day 9 of life showed a reduction in the size of the thrombus. This suggested that anticoagulant therapy was effective, and the dose of enoxaparin was gradually adjusted to 2.1 mg/kg twice a day to achieve the target anti-Xa level (Figure 4).

**Figure 5:**
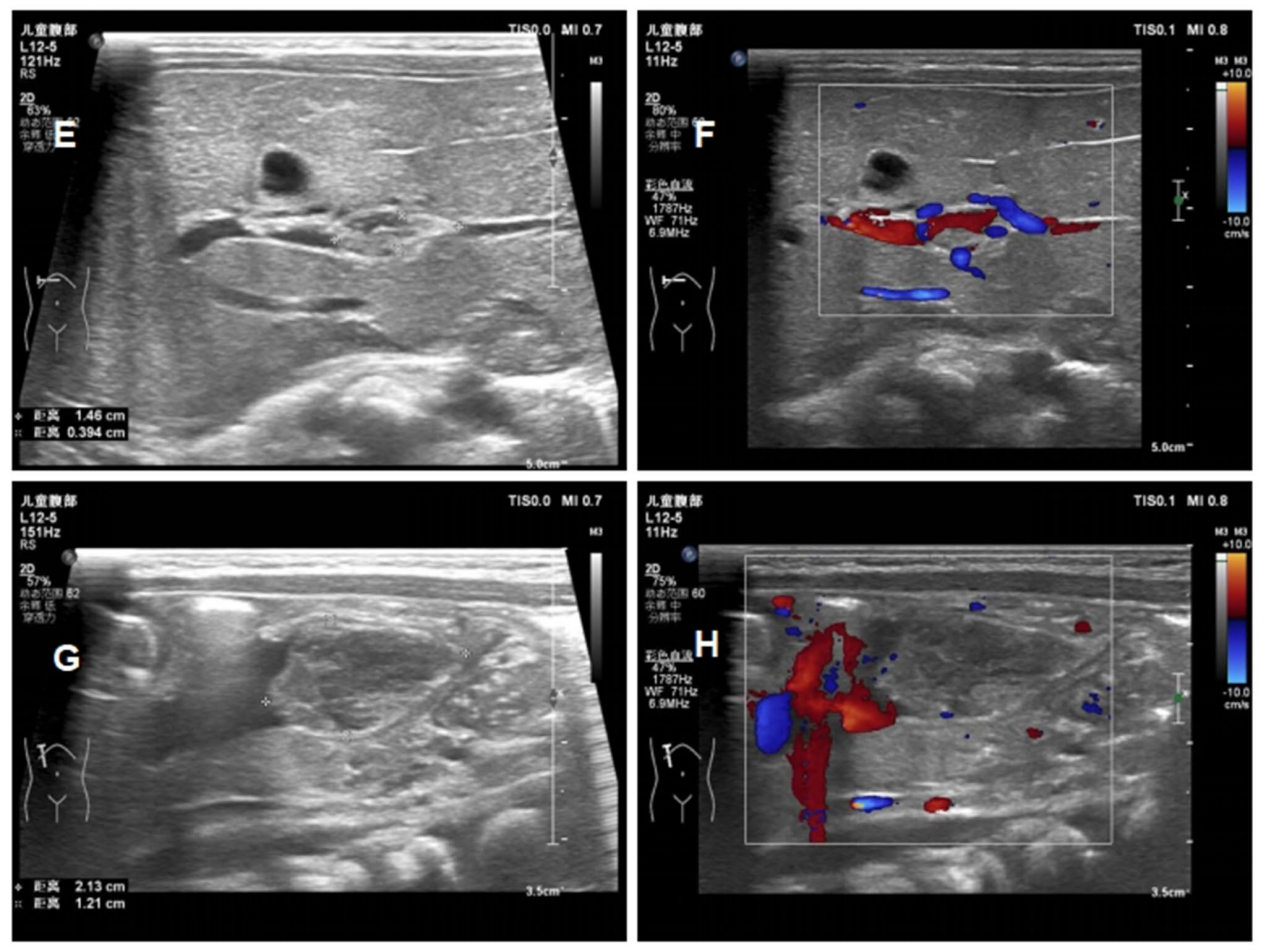
Ultrasound images of the patients ont day 16 of life **(E)** reduction in the size of the portal vein thrombus at day 16; **(F)** the blood flow signal of portal vein thrombosis at day 16; **(G)** reduction in the size of the umbilical vein thrombus at day 16; **(H)** the blood flow signal of umbilical vein thrombosis at day 16.

At day 16 of life, a fourth ultrasound showed that the thrombus was significantly smaller than before, and the stenosis degree of the portal vein decreased from 75 to 30% (Figure 5). Laboratory tests showed that Protein C had risen to 40%. Therefore, the baby was discharged in good clinical condition and with enoxaparin (2.1 mg/kg q12h) to be continued Table 2 shows the fetal hematology test indicators and Table 3 for pregnant woman.

**Table 2:** Fetal hematology test indicators.

Coagulation	Reference range	Age 1d	Age 9d	Age 12d	Age 20d	Age 32d	Age 2 m
PT(s)	12–15	18	12.8	13	12.8	12.9	12.6
INR	0.85–1.15	1.52	1.01	1.03	1.01	1.02	0.99
APTT(s)	30–45	51	50.40	65.2	86.9	68.1	46.5
TT(s)	<21	16.9	19.8	35.70	43	31.7	19.2
FIB(g/L)	2–4	2	1.94	2.03	1.91	1.66	1.55
D-Di (μg/ml)	0–0.5	2.38	1.28		0.54	0.3	<0.22

**Table 3:** Hematology test indicators for pregnant women.

Coagulation	Reference range	30 weeks pregnant	37 weeks pregnant	39 weeks pregnant
PT(s)	12–15	11.8	12.1	11.8
INR	0.85–1.15	0.94	0.93	0.90
APTT(s)	30–45	32.7	32.8	33.4
TT(s)	<21	16.0	16.5	15.6
FIB(g/L)	2–4	4.37	4.49	4.46

**Figure 6:**
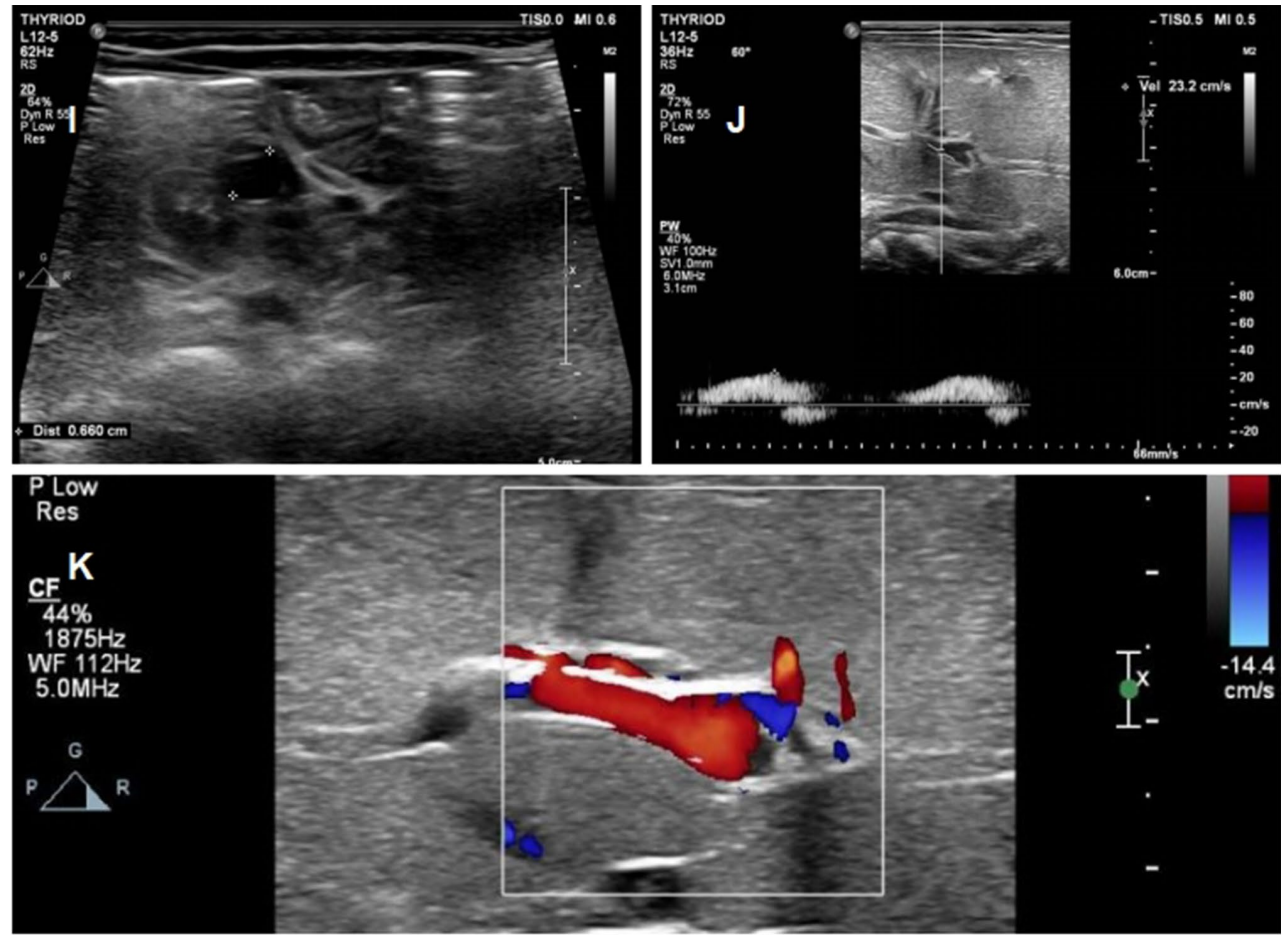
Ultrasound images of the patients on day 26 of life **(I)** Complete regression of the umbilical vein thrombus at day 26; **(J)** Complete regression of the portal vein thrombus at day 26; **(K)** normal blood flow signal of portal vein.

The baby was kept under regular follow-up with a plan for clinical assessment and ultrasonography every week. Another ultrasound examination at day 26 of life, showed an almost complete resolution of the thrombus (Figure 6). Enoxaparin was suspended at day of life 33, with a 30-day course of treatment. During her treatment, a routine cerebral ultrasound did not show periventricular or intraventricular hemorrhage following the administration of LMWH therapy, and routine blood examinations performed weekly showed no heparin-induced thrombocytopenia (HIT). Subsequently, the baby was followed up once a month. She was in excellent clinical conditio and had no recurrence of thrombosis at the 10 month follow-up.

The inspection results for the newborn (hospitalized from 2020.4.23 to 5.5) show that the NTSH, NPKU, N17OH, and NG6PD levels are within the normal ranges. The eye and hearing screenings were also normal. The blood routine test showed slightly elevated levels of white blood cells and neutrophil ratio but no other abnormalities. The blood biochemistry test showed normal liver and renal function, with slightly elevated total bilirubin levels. The skull B-ultrasound did not detect any abnormalities. The cardiac ultrasound showed a patent foramen ovale, and the abdominal ultrasound showed no abnormalities in the liver, gallbladder, spleen, pancreas, or kidneys. As for the mother (hospitalized from 2022.4.21 to 4.25), her blood biochemistry showed slightly elevated levels of total protein and potassium, while the blood routine test showed slightly elevated levels of white blood cells and neutrophil ratio.

## Discussion and conclusions

Umbilical vascular thrombosis is a rare pregnancy complication, that is closely associated with increased perinatal morbidity and mortality. A large number of clinical studies have confirmed that the formation of thrombosis in umbilical vessels can lead to fetal distress, fetal growth restriction, stillbirth, hypoxic–ischemic encephalopathy and cerebral palsy (5–8). According to statistics, venous, venous and arterial, and arterial thrombosis occur in 70, 20, and 10% of cases, respectively (2). Thus, UVT occurs more frequently than umbilical artery thrombosis (UAT).

The pathogenesis and mechanism of umbilical vascular thrombosis are not yet clear. Hypercoagulability, blood fow stasis and endothelial damage are three key elements of thrombosis. Hypercoagulability may be associated with inherited or acquired thrombophilia. More than 60% of idiopathic thrombotic events during pregnancy have been reported to be related to inherited thrombophilia (9). Anatomical abnormalities (i.e., anomalous insertion, abnormal coiling, abnormal length, or umbilical cord stricture) as well as mechanical injury of the cord (i.e., compression, knots, torsion) have been confirmed as high risk factors to induce flow stasis and thrombosis of the umbilical vessels (10, 11). The presence of endothelial damage may be due to intrauterine infections and umbilical vein catheterization leading to cord thrombosis (12, 13). In addition, umbilical vascular thrombosis may also be related to maternal pathology such as maternal diabetes, hypertension and smoking (14).

In our case, no anatomical cord anomaly or umbilical cord torsion was noted, and tests for thrombophilias in the baby revealed no evidence of inherited thrombophilia or acquired conditions associated with UVT, and the parents did not have any identifiable risk factors warranting investigation for inherited disorders. The only associated risk factor we found was maternal gestational diabetes mellitus. According to Fritz’ s hypothesis, unstable maternal blood glucose levels can lead to imbalanced expression of endothelial vasodilatation factors and shrinkage factors, which can induce dysfunction of blood coagulation and the eventual occurrence of thrombosis (15).

The prenatal diagnosis of UVT remains a clinical challenge, and ultrasound remains the optimal imaging modality, which may provide significant indications in prenatal screening since it can recognize abnormalities (16). In our case, we report a 33-year-old woman with umbilical vein thrombosis at 21 weeks gestation who was diagnosed by ultrasound. During the second and third trimestesr, the hyperechoic segment in the umbilical vein gradually enlarged, but there were no obvious abnormalities in umbilical cord blood flow, fetal development or fetal heart rate monitoring. After delivery portal vein thrombosis was also found. It has been reported that umbilical venous catheter placement is the major risk factor for portal vein thrombosis (17). Considering that no umbilical vein catheterization was performed, we suspect that the umbilical vein thrombus embolized to the portal vein. The baby was in good clinical condition, without growth restriction, and it is possible that the umbilical vein was not completely blocked by the thrombus. The oxygen and nutrition supply of the umbilical vein were not completely interrupted, the thrombosis was limited to the umbilical vein, and the portal vein and inferior vena cava were not involved in the uterus.

Umbilical vein thrombosis is a rare pregnancy complication that leads to poor fetal outcomes. Umbilical cord anomalies, abnormal fetal coagulation function, intrauterine infection, and maternal diabetes could be likely etiologies. Although ultrasound remains the most reliable diagnostic tool, thrombi involving the umbilical vein may not be accompanied by abnormal blood flow signals, which are easily missed in the antepartum period. Furthermore, sudden fetal death could occur without any foretelling signals. We should pay more attention to unusual clinical symptoms, such as decreased fetal movement or abnormal fetal heart rate monitoring.

This case report provides novel information regarding the diagnosis and clinical course of a rare case of umbilical vascular thrombosis (UVT). It highlights the importance of antenatal screening through ultrasound imaging to identify potential risk factors, such as maternal gestational diabetes mellitus, and close monitoring of fetal development throughout pregnancy. The report emphasizes the role of hypercoagulability, blood flow stasis, and endothelial damage in the pathogenesis of UVT, and suggests that not all cases of UVT lead to poor fetal outcomes. We emphasize the need for further research to fully understand the mechanisms of UVT, which may improve prenatal diagnosis and management strategies. Overall, this case report provides valuable insights into the diagnosis and management of UVT, particularly in high-risk pregnancies, and underscores the importance of maintaining a high level of clinical suspicion for this rare but serious complication.

## Data availability statement

The original contributions presented in the study are included in the article/supplementary material, further inquiries can be directed to the corresponding author.

## Ethics statement

The studies involving humans were approved by Medical Ethics Committee of Yuying Children's Hospital Affiliated to Wenzhou Medical University. The studies were conducted in accordance with the local legislation and institutional requirements. The participants provided their written informed consent to participate in this study. Written informed consent was obtained from the minor(s)’ legal guardian/next of kin for the publication of any potentially identifiable images or data included in this article.

## Author contributions

W-WD: Investigation, Methodology, Writing – original draft, Data curation, Project administration. Q-SH: Investigation, Project administration, Writing – review & editing. L-HY: Methodology, Validation, Writing – review & editing. S-QC: Writing – review & editing, Investigation, Validation. J-FY: Conceptualization, Data curation, Project administration, Resources, Supervision, Validation, Visualization, Writing – review & editing.
